# Compression benchmarking of holotomography data using OME-Zarr format

**DOI:** 10.1371/journal.pone.0351560

**Published:** 2026-07-08

**Authors:** Dohyeon Lee, Juyeon Park, Juheon Lee, Chungha Lee, YongKeun (Paul) Park

**Affiliations:** 1 Department of Physics, Korea Advanced Institute of Science and Technology (KAIST), Daejeon, Republic of Korea; 2 KAIST Institute for Health Science and Technology, KAIST, Daejeon, Republic of Korea; 3 Tomocube Inc., Daejeon, Republic of Korea; PLOS, UNITED KINGDOM OF GREAT BRITAIN AND NORTHERN IRELAND

## Abstract

Holotomography (HT) is a label-free, three-dimensional quantitative phase imaging technique that captures refractive index distributions of biological samples at sub-micron resolution. As modern HT systems enable high-throughput and large-scale acquisition, they produce terabyte-scale datasets that require efficient data management. This study presents a systematic benchmarking of data compression strategies for HT data stored in the OME-Zarr format, a cloud-compatible chunked data structure suitable for scalable imaging workflows. Using six representative datasets from five biological samples, we evaluated combinations of preprocessing filters and 13 compression algorithms across multiple compression levels. Performance was assessed in terms of compression ratio, bandwidth, and decompression speed. A throughput-based evaluation metric was introduced to capture realistic performance under varying network constraints, revealing that the optimal compression strategy is strongly dependent on available system bandwidth. Across a wide range of bandwidth conditions, Pcodec consistently exhibited the most balanced overall performance, followed by Blosc-zstd and zstd. The results offer practical guidance for the storage and transmission of large HT datasets and serve as a reference for implementing scalable, FAIR-aligned imaging workflows in cloud and high-performance computing environments.

## 1. Introduction

Holotomography (HT), also known as three-dimensional (3D) quantitative phase imaging, has emerged as a powerful, label-free, high-resolution imaging modality. HT reconstructs the 3D refractive index (RI) distribution of unlabeled live biological samples from multiple two-dimensional light-field measurements, analogous to X-ray computed tomography [1–6]. Its quantitative, tomographic, and noninvasive imaging capabilities have enabled broad applications across cell biology [7,8], biophysics [9,10], neuroscience [11], microbiology [12], immunology [13], regenerative medicine [14–16], and histopathology [17].

Recent advances in optical systems and reconstruction algorithms have significantly improved the speed and scalability of HT, making it suitable for high-throughput applications. State-of-the-art HT systems can acquire a 3D image volume of 200 μm × 200 μm × 150 μm at spatial resolutions of 150 nm × 150 nm × 800 nm within 1 second. Lateral stitching techniques now enable imaging of centimeter-scale tissue sections [17], while robust long-term imaging allows time-lapse acquisition spanning several weeks [18]. Moreover, the recent development of birefringence-sensitive HT systems enables the reconstruction of 3 × 3 tensor RI representations at each voxel, facilitating a deeper understanding of molecular alignment and anisotropic structures in biological samples [19–23].

**Fig 1:**
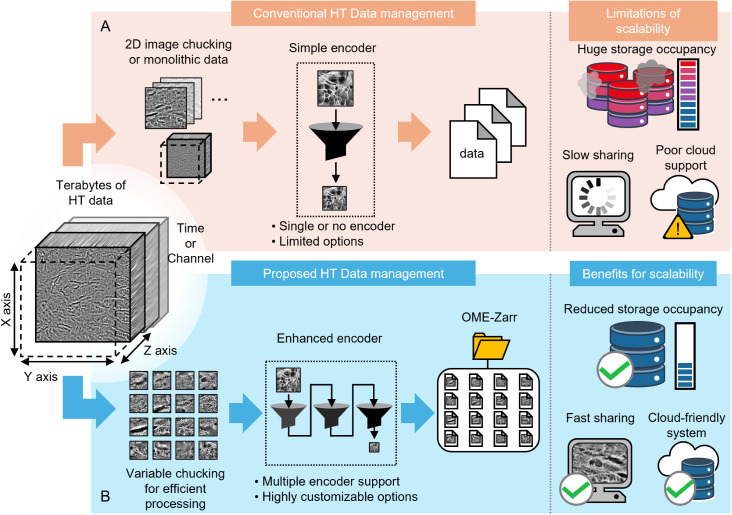
Comparison of conventional and proposed data management strategies for holotomography (HT) imaging. A, Conventional methods use simple or no encoding with limited chunking, resulting in large storage needs and poor scalability. B, The proposed approach employs variable chunking, customizable encoders, and OME-Zarr format, enabling efficient storage, fast sharing, and cloud compatibility.

However, the ability of HT to generate extremely large datasets over extended time periods introduces substantial challenges in data storage, transmission, and real-time accessibility (Fig 1). Traditional image formats such as TIFF, although structured, are limited in scalability, metadata management, and cloud compatibility due to their monolithic file structures and lack of efficient chunking or encoding strategies (Fig 1A). HDF5, while supporting multi-dimensional arrays, suffers from limited support for real-time or remote access, as entire datasets typically need to be downloaded prior to analysis [24,25].

Regarding compression, several approaches have been proposed for 3D imaging, such as hybrid entropy coding [26], learning-based methods [27,28], and domain-specific representations [29]. These works primarily highlight the advantages of specific algorithms, whereas a comprehensive and application-oriented benchmarking framework remains lacking.

To overcome these limitations, Zarr, a cloud-native, chunked, and compressed data storage format, has gained attention as a promising alternative for managing terabyte-scale imaging data [25,30]. Zarr divides data into user-defined chunks that can be processed and stored independently, enabling parallel read/write operations, distributed computing, and seamless integration with cloud infrastructure. Users can define custom chunking strategies for their specific needs and optimize performance. Before converting a chunk of data into a file, it can be processed through various encoding options, including preprocessing filters and compression algorithms, allowing for efficient storage utilization and faster data sharing. The OME-Zarr specification extends this format for multi-dimensional bioimaging by incorporating standardized metadata for dimensionality, resolution, and channel information, thereby supporting complex imaging workflows in scalable and FAIR (Findable, Accessible, Interoperable, and Reusable)-compliant environments (Fig 1B) [31].

Despite the advantages of Zarr-based storage, the selection of an optimal compression strategy remains nontrivial. Efficient encoding not only reduces storage footprint but also directly impacts data access speed—an increasingly critical factor in AI-driven analysis, real-time processing, and cloud-native applications [32]. Although prior studies have reported compression benchmarks in other imaging contexts [33–35], a comprehensive evaluation tailored to HT imaging has not been performed.

In this study, we present a systematic benchmarking analysis of compression strategies for HT data stored in OME-Zarr format. We assess the effects of preprocessing filters (bit-depth rounding and byte shuffling) and compare the performance of widely used compression algorithms, including gzip, LZMA, bzip2, zlib, LZ4, Snappy, zstd, Pcodec, zfp, and Blosc-based methods [36–45]. Our evaluation spans diverse datasets, including both conventional and birefringent HT volumes, across conditions of varying spatial density and signal strength. We measure and compare compression ratio, compression bandwidth, and decompression bandwidth under realistic usage scenarios, including simulated cloud-based access.

To provide practical guidance for diverse computational environments, we introduce a throughput-based evaluation metric that integrates network transfer bandwidth with compression and decompression speeds. This metric enables a context-aware comparison of compression strategies, helping users select optimal configurations tailored to specific storage and data transfer conditions.

The throughput-based benchmark shows that optimal compression choices depend on available bandwidth: higher compression ratios are advantageous in low-bandwidth scenarios, while faster (de)compression dominates performance at high bandwidth. Among all tested configurations, Pcodec provided the most balanced overall performance.

Our findings offer key insights for scalable, high-performance HT data management, supporting efficient storage, rapid access, and smooth integration with modern computational pipelines. This work provides a foundational resource for HT imaging workflows and contributes to broader efforts in building FAIR-aligned data infrastructures for large-scale biomedical imaging.

## 2. Methods

### 2.1. Benchmarking process overview

**Fig 2:**
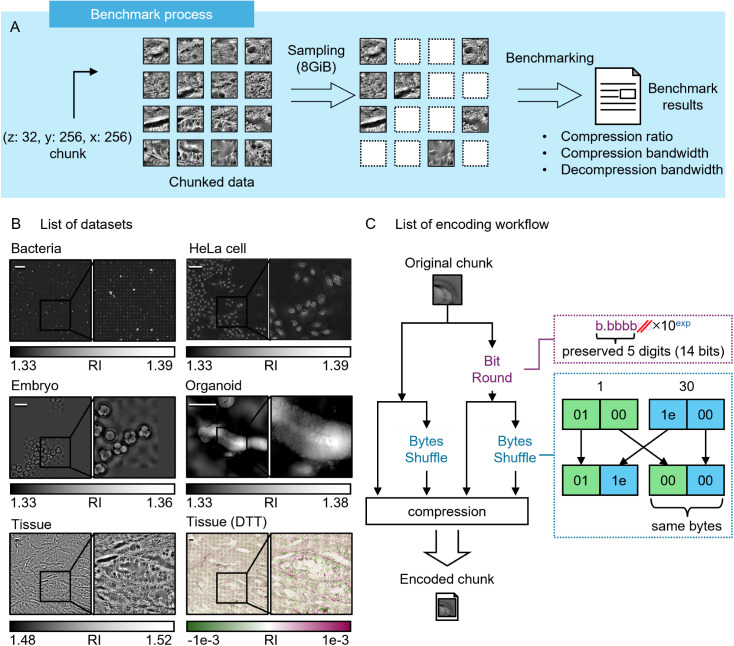
Benchmarking workflow for HT data compression. A, Overview of the benchmarking process with fixed chunk shape and sampling size (8 GiB). B, Representative datasets used in the benchmark. Scale bar = 100 μm. C, Encoding workflow illustrating applied filters, including bit rounding and byte shuffling, followed by various compression options.

To systematically assess compression performance for HT data, we designed a benchmarking workflow based on chunked OME-Zarr datasets (Fig 2a). All datasets were stored in single-precision floating-point format, and the chunk size was fixed at 32 × 256 × 256 along the z, y, and x dimensions, respectively. This configuration ensured a uniform data structure across all test cases, with each uncompressed chunk occupying exactly 8 MiB of memory.

Due to the large volume of the original HT datasets, benchmarking was performed on a sampled subset totaling 8 GiB from each dataset, corresponding to 1024 randomly selected chunks. This random sampling strategy captures spatial diversity by selecting chunks from different locations across the dataset, thereby covering a range of structural characteristics without requiring an exhaustive and time-consuming search. Although this approach does not encompass all possible data patterns, it provides sufficiently representative coverage for efficient benchmarking. For each compression configuration, we evaluated three core performance metrics: compression ratio, compression bandwidth, and decompression bandwidth, enabling a comprehensive analysis of both storage efficiency and computational throughput.

Benchmarks were executed in a multi-threaded environment to reflect common usage patterns in OME-Zarr pipelines, which typically leverage parallel I/O and chunk-level operations. Consequently, the reported bandwidth metrics represent peak throughput under full CPU utilization. To isolate computational performance from potential disk I/O bottlenecks, all compression and decompression tasks were performed entirely in memory. Input data chunks were preloaded into RAM, compressed data was written to separate memory buffers, and decompression was conducted by reading directly from those buffers. This design ensured that the measurements reflect true processing performance, unaffected by storage latency.

All tests were conducted on a high-performance workstation equipped with an AMD Ryzen Threadripper 2950X processor (32 threads) and 128 GB of DDR4 RAM, providing ample parallel computing capacity for multi-threaded compression workloads. This setup ensured reproducibility and stability during benchmark execution across all tested configurations.

The three bandwidth regimes (low: 100–500 MB/s, mid: 1–5 GB/s, high: > 10 GB/s) were chosen to approximate real-world environments: campus or departmental networks (1 GbE ≈ 125 MB/s, 10 GbE ≈ 1.25 GB/s), HPC cluster interconnects (25–100 GbE, > 3–12 GB/s), and workstation-to-S3 transfers over WAN, where effective throughput often falls in the low regime due to latency and protocol overheads.

### 2.2. Dataset preparation

Six representative holotomographic datasets were acquired from five types of biological samples: Bacteria, HeLa cells, organoids, embryos, and tissue (Fig 2b). These samples cover common imaging scenarios in HT of single-cell to multicellular imaging [1]. We summarized the source, imaging setup used, and properties of each dataset in Table 1.

**Table 1:** List of used biological samples and properties of raw data and used imaging setup.

Sample name		Bacteria	HeLa cell	Organoid	Embryo	Birefringent tissue
Source of data		[46]	[47]	[14]	This work	This work
Imaging setup		HT-2H, Tomocube	HT-X1, Tomocube	HT-X1, Tomocube	Custom setup	Custom setup
Uncompressed data size (GiB)		16.0	258.6	501.9	2885.5	286.7
Shape	Time	1	73	289	161	1
	Channel	1	1	1	1	9
	Z	64	66	138	116	80
	Y	8192	3796	1838	6440	10807
	X	8192	3796	1838	6440	9890
Resolution	Time (s)	.	1200	600	30	.
	Z (nm)	360	950	1,050	1,080	400
	Y (nm)	110	160	160	210	100
	X (nm)	110	160	160	210	100

For the generalizability of our approach across various HT implementations, we include data from three different imaging modalities. Each imaging setup employs distinct optical systems and reconstruction schemes. HT-2H uses a laser-based interferometric system, while HT-X1 employs a spatially incoherent light source and non-interferometric reconstruction that eliminates speckle noise. Embryo imaging was performed using a custom-built setup employing a 624 nm light source with carefully controlled illumination intensity to ensure embryo-compatible acquisition conditions, while operating on the same fundamental imaging principles as commercial HT systems [16]. In contrast, the birefringent tissue data acquired in this study were obtained using a custom-built HT microscope specifically designed for birefringence-sensitive imaging, as current commercial systems do not support birefringence measurements [19,48].

This dataset is represented as a 3 × 3 refractive index tensor, which includes the refractive index values that can be measured by conventional HT along with the shear components associated with birefringent refractive indices. These shear components typically exhibit low signal amplitudes and are more susceptible to noise, thereby providing a stringent testbed for evaluating compression performance on weak, low-contrast signals.

The raw HT data from Tomocube’s setups only preserve values up to four decimal places. To simulate the raw acquisition noise typically present in unprocessed floating-point HT data, uniform random noise in the range of –5 × 10 ⁻ ⁵ to 5 × 10 ⁻ ⁵ was added to each voxel before compression benchmarking. This approach ensures an evaluation of compression performance in which noise is retained.

For conventional in vitro fertilization, 10-week-old BALB/c females and 8-week-old BALB/c males were obtained from Orient Bio Inc. (Sungnam, South Korea). The animals were provided with rodent chow and reverse osmosis water. All experimental procedures on embryos were conducted in accordance with the Institutional Animal Care and Use Committee (IACUC) guidelines (KA2023–005-v4). For the collection of gametes required for in vitro fertilization, mice were euthanized by trained personnel using cervical dislocation without anesthesia, an immediate and minimally distressing method. This procedure was chosen in consideration of previous reports that CO_2_ inhalation can cause physiological disturbances, such as hypothermia and blood pH alterations, which may adversely affect gamete quality [49]. To minimize unnecessary suffering, the minimum number of animals required for the experiments was used.

The tissue samples consisted of formalin-fixed, paraffin-embedded (FFPE) colon cancer tissues collected from Asan Medical Center. Ethical approval was granted by the Institutional Review Board (IRB) with a waiver of informed consent (IRB No. 2021−1698). The tissue data used in this study were accessed on 17 October 2024 for research purposes. The authors did not have access to any personal records of participants during or after data collection. All procedures conformed to the ethical standards outlined in the Declaration of Helsinki [50].

### 2.3. Filter and compression algorithms for encoding data

In the encoding pipeline, raw data were first processed using one or more preprocessing filters, followed by compression using a selected algorithm (Fig 2c). We systematically explored all possible combinations arising from two types of filters and 13 distinct compression algorithms across various compression levels. We selected processing filters and compression algorithms from Zarr’s official codec package, Numcodecs, with Snappy incorporated via the Imagecodecs package [51,52]. Using official and well-established packages ensures the reproducibility and robustness of this work.

Two preprocessing filters were evaluated: bit rounding and byte shuffling. These filters enhance compressibility by restructuring the data layout to expose redundancy. Bit rounding reduces the numerical precision of floating-point values by setting less significant bits to zero [53]. While this introduces minor information loss, it significantly reduces entropy, improving the effectiveness of downstream compression. Although bit rounding is a lossy preprocessing step, it is fully compatible with conventional lossless compression algorithms and allows numerical precision to be explicitly controlled. We configured bit rounding to retain four significant digits, and the corresponding information loss is illustrated in S1 Fig. The byte shuffling filter reorganizes bytes within the data so that similar byte positions across adjacent values are grouped. This transformation increases local redundancy and improves pattern detection by compression algorithms, thereby boosting compression efficiency without loss of precision.

Following the filtering step, datasets were compressed using 13 algorithms encompassing both classical and speed-enhanced compression algorithms (Table 2). The classical algorithms—gzip, zlib, bzip2, and LZMA—rely on dictionary-based encoding followed by classical entropy coding schemes such as Huffman coding and arithmetic coding [37–40]. These algorithms typically provide high compression ratios; however, their complex encoding procedures result in increased computational cost. These well-established algorithms serve as a useful benchmark for evaluating newer alternatives.

**Table 2:** List of compression algorithms and levels used in benchmarking.

Type	Compressor name		Levels		
			(L)ow	(M)edium	(H)igh
Classical		gzip	1	5	9
		zlib	1	5	9
		bzip2	1	5	9
		LZMA	1	5	9
Speed-enhanced		LZ4	.	.	1
		Snappy	.	.	**0
		zstd	1	11	22
		*Pcodec	1	6	12
	Blosc	zlib	1	5	9
		LZ4	1	5	9
		zstd	1	5	9
Error-bounded	*zfp	lossless	.	.	**0
		lossy	.	.	**0

Numeric values denote algorithm-specific compression settings corresponding to Low (L), Medium (M), and High (H) levels. *Pcodec and zfp are not compatible with the Shuffling filter due to the unsupported output type (uint8). ** Snappy and zfp have fixed compression levels.

In contrast, speed-enhanced algorithms are designed for faster processing through lightweight implementation, novel algorithm types, or hardware-aware design. LZ4 and Snappy skip the time-consuming entropy coding stage to achieve high speed at the expense of compression ratio [41,45]. Zstd and Pcodec adopt the asymmetric numeral system entropy coding algorithm [36,42]. They offer high compression ratios while maintaining fast throughput, making them strong candidates for real-time or cloud-based workflows. We also evaluated the Blosc meta-compression framework, which divides datasets to optimize CPU cache usage and reduce memory bus activity. Blosc was tested with three backend compressors: zlib, LZ4, and zstd [44]. For a scientific lossy compressor, we tested zfp in both lossless mode (error bound = 0) and lossy mode (error bound < 10 ⁻ ^4^) [43,54]. The lossy mode is similar to bit rounding in that it allows controlled precision reduction.

To assess how varying compression aggressiveness impacts performance, each algorithm was tested at three representative compression levels. The low level prioritizes speed and minimal overhead, the medium level aims to balance speed with reasonable data reduction, and the high level seeks maximum compression ratio at the expense of processing time. For most algorithms, these correspond to levels 1 (L), 5 (M), and 9 (H). For zstd, which supports a wider compression range, levels 1, 11, and 22 were used. Pcodec was tested at levels 1, 6, and 12. LZ4, designed for ultra-fast compression, was evaluated at its single high-speed setting (level 1), which remains faster than other algorithms across comparable configurations. Snappy and zfp have fixed compression levels, as indicated in Table 2.

This comprehensive setup allowed for a detailed analysis of how different encoding strategies interact with filter combinations and system bandwidth, providing a robust framework for optimizing compression in holotomography imaging workflows.

## 3. Results

### 3.1. Evaluation of compression ratio

**Fig 3:**
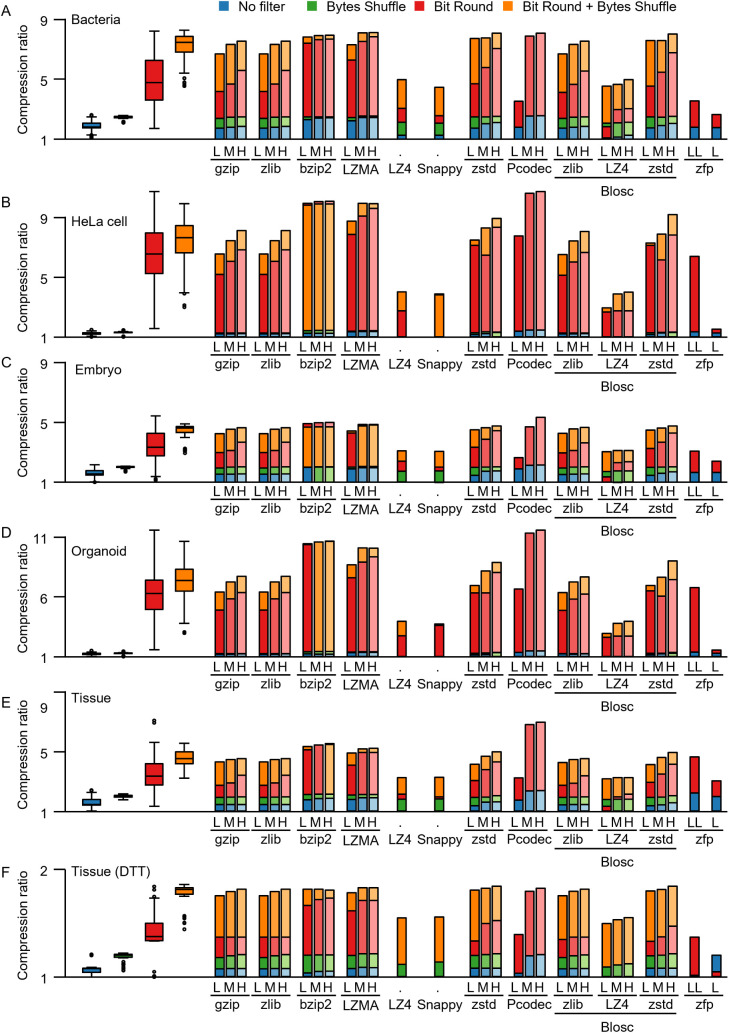
Compression ratio comparison across datasets and filtering methods. Left panels show average compression ratios for each filter: No filter (blue), byte shuffle (green), bit round (red), and combined bit round + byte shuffle (orange). Overlaid bars represent results for each compression algorithm tested at low **(L)**, medium **(M)**, and high (H) compression levels. For the zfp algorithm, LL denotes lossless mode and L denotes lossy mode.

To evaluate the effects of various filtering and compression strategies on HT data, we analyzed the overall compression ratio across different sample types, preprocessing filters, and compression algorithms (Fig 3). The left panel of each plot in Fig 3 presents the average compression ratios for four filtering configurations: no filter, byte shuffle, bit rounding, and a combination of bit rounding and byte shuffle. The right panels display the full distribution of compression outcomes, providing a more detailed view of algorithm-specific performance under different conditions.

Across all datasets, filtering improved compression efficiency by increasing data regularity and redundancy. Bit rounding consistently yielded higher compression gains than byte shuffling alone. This is because bit rounding eliminates scientifically insignificant bits by setting them to zero, introducing highly redundant zero-value regions without compromising meaningful information. Since many scientific measurements are limited by the precision of the instrument, reducing excess precision in this way is often justifiable and effective. In contrast, byte shuffling preserves all information while reorganizing byte order within chunks to make patterns more detectable by compression algorithms. This technique is most effective when adjacent voxel values are similar or change gradually—conditions commonly found in HT images due to the inherently slow spatial variation in biological samples, which are also leveraged during HT reconstruction [55].

Thus, while byte shuffling preserves all information (lossless), bit rounding introduces controlled loss (lossy) for improved compression. The optimal filtering strategy depends on whether strict data fidelity is required. Table 3 summarizes the recommended filtering combinations based on the desired compression type.

**Table 3:** Recommended filter combinations for lossless and lossy compression of holotomographic data.

	Lossless	Lossy
Best filter combination	Bytes Shuffle	Bit Round + Bytes Shuffle

Across most algorithms, the compression ratio followed a consistent trend: no filtering < byte shuffle < bit rounding < bit rounding + byte shuffle. However, certain exceptions were observed for specific algorithms such as LZMA, bzip2, and LZ4, due to their distinct internal compression mechanisms. For instance, LZMA relies on long-range dictionary matching, which byte shuffling can disrupt [39], while bzip2’s Burrows–Wheeler transform has a reordering step and may conflict with byte shuffling [37,56]. LZ4, optimized for short-range pattern detection, benefited more from byte shuffling than from bit-rounding in noise-sensitive shear component of tissue refractive index data [41].

Pcodec achieved results comparable to other compressors with byte shuffling. This is likely due to its internal preprocessing that maps values to latent variables suitable for entropy encoding. Zfp exhibits atypical behavior: applying lossless zfp to bit-rounded data yields higher compression ratios than its lossy mode across all datasets.

Shear components from birefringent tissue data show lower compression ratios than other datasets (Fig 3). The reduction is likely due to their smaller, more variable values and higher noise susceptibility. These conditions increase entropy and reduce redundancy, limiting the effectiveness of compression. These findings suggest that further improvements in compressibility for noise-prone datasets could be achieved through additional preprocessing, such as denoising or more aggressive precision reduction beyond standard bit rounding. Future work may explore these options to improve storage efficiency without compromising data utility in downstream analyses.

### 3.2. Evaluation of compression and decompression bandwidth

Beyond compression ratio, the bandwidth of compression and decompression plays a pivotal role in determining the practical efficiency of a compression strategy. While higher compression ratios enhance storage efficiency, suboptimal speed performance can introduce significant computational bottlenecks, especially in real-time processing or latency-sensitive workflows. The utility of HT data lies not only in how compactly it can be stored but also in how quickly it can be accessed and processed.

For instance, decompression speed directly impacts the responsiveness of downstream tasks, such as quantitative analysis, 3D visualization, and AI inference. In cloud-based HT workflows—where reconstruction and analysis are performed on remote servers—the combination of compression and decompression bandwidth dictates how quickly results can be transmitted to and accessed by end users. These scenarios illustrate the need to optimize both bandwidth and compression efficiency to ensure a seamless user experience and effective integration into high-throughput, distributed imaging pipelines.

**Fig 4:**
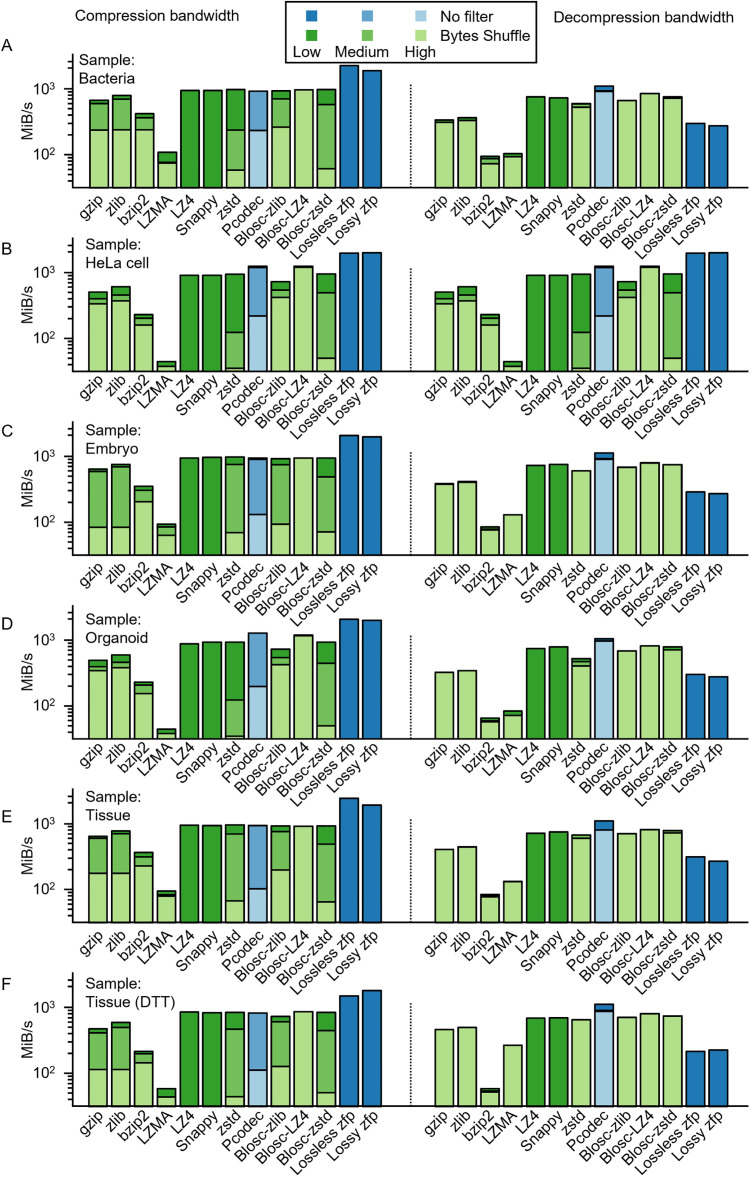
Compression and decompression speed benchmarks with byte shuffle enabled. Left panels show compression bandwidths, and right panels show decompression bandwidths, measured in mebibytes per second (MiB/s). Values above each bar indicate relative speed compared to gzip, averaged across compression levels.

To isolate bandwidth performance from compression ratio effects, we conducted speed benchmarks using byte shuffling only and no filtering option for Pcodec and zfp, as this filtering strategy showed the most stable and consistent compression ratios across datasets (Fig 4). All benchmarks were executed in a simulated parallel compression environment to reflect realistic usage of chunked storage in OME-Zarr, where parallelized processing is often leveraged. As expected from the general trade-off between compression ratio and speed, algorithms achieving higher compression ratios in Fig 3 tended to exhibit lower bandwidths, whereas speed-optimized algorithms showed the opposite trend.

The compression bandwidth results reveal that modern, speed-optimized algorithms and error-bounded algorithms such as LZ4, Snappy, zstd, Pcodec, zfp, and Blosc-based variants exhibit superior performance, particularly at low compression levels. These algorithms achieve significantly higher throughput compared to traditional methods such as gzip, bzip2, or LZMA. However, their performance advantage diminishes as the compression level increases. For example, at high compression levels, zstd’s compression bandwidth becomes comparable to, or even lower than, that of conventional methods. This suggests a trade-off: speed-optimized algorithms incur increasing computational overhead at higher compression ratios, potentially offsetting their usual performance benefits. Therefore, both the choice of compression algorithm and its configuration level must be considered in tandem to optimize processing speed.

In contrast, decompression benchmarks showed consistent superiority of speed-enhanced algorithms across all datasets and compression levels. Regardless of configuration, LZ4, zstd, and Blosc-based methods demonstrated significantly higher decompression bandwidths compared to classical algorithms. This indicates that speed-enhanced compressors are better engineered for fast data restoration—an especially valuable trait in scenarios where rapid access to compressed data is critical.

### 3.3. Definition of a throughput-based metric for HT compression evaluation

Since no single benchmark parameter can independently determine the optimal compression strategy, we introduce a unified, throughput-based evaluation metric designed to reflect the practical demands of future HT data workflows. Modern data management in HT extends beyond simple storage and sharing, increasingly functioning as a platform for reconstruction, visualization, and multidimensional analysis. In this evolving landscape, it is anticipated that users will offload computationally intensive tasks—such as 3D reconstruction and polarization analysis—to high-performance cloud or server-based platforms [57,58].

Our proposed metric models a typical cloud-based HT reconstruction and analysis scenario, leveraging the chunk-level parallelism supported by the OME-Zarr format. Unlike traditional file-based workflows, where compression, transfer, and decompression times are additive, chunked parallel workflows are constrained by the slowest of these operations. Thus, our metric focuses on identifying the bottleneck step and quantifying the system’s maximum sustainable throughput, offering a realistic measure of overall performance in distributed HT imaging pipelines.

We define the effective bandwidth (EB) as the product of the compression ratio (CR) and the available transfer bandwidth (TB), representing the logical throughput gain achieved via compression:


$$\begin{array}{c} \text{EB}=\text{CR}\times \text{TB}. \end{array}$$ (1)

Here, CR is computed as the average compression ratio obtained using either byte shuffling alone or a combination of byte shuffling and bit rounding, thus capturing both lossless and lossy compression scenarios. This average allows for an inclusive evaluation that accounts for precision-sensitive and precision-tolerant applications.

Next, we define the maximum achievable bandwidth (B_max_) as the minimum value among the effective bandwidth, compression bandwidth (CB), and decompression bandwidth (DB):


$$\begin{array}{c} {\text{B}}_{\text{max}}=\text{min}(\text{EB},\text{CB},\text{DB})=\text{min}(\text{CR}\times \text{TB},\text{CB},\text{DB}). \end{array}$$ (2)

This formulation reflects the fact that system throughput is inherently limited by the slowest stage in the compression–transfer–decompression pipeline. Therefore, B_max_ serves as a unified indicator of end-to-end performance and provides a data-driven basis for selecting compression strategies suited to real-world HT data management scenarios, particularly in cloud-based and remote processing environments.

This metric provides a systematic basis for selecting the optimal compression method under varying data transfer constraints, making it particularly relevant for high-throughput HT imaging applications in cloud-based environments.

### 3.4. Application of the metric across varying network bandwidths

**Fig 5:**
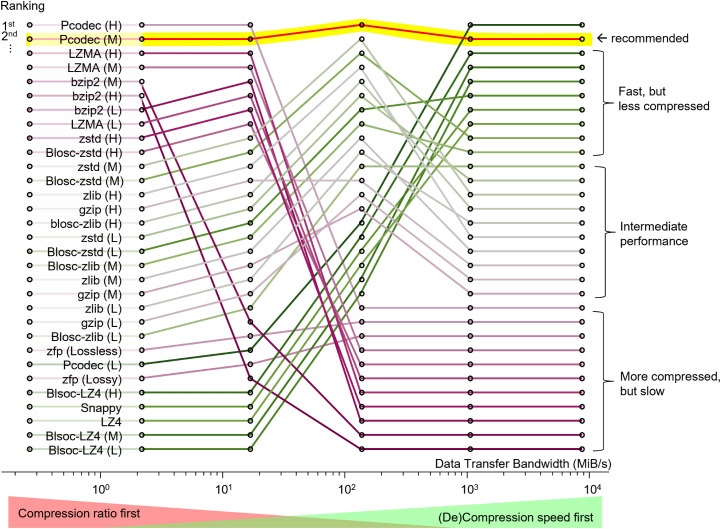
Ranking of compression methods based on a throughput-optimized metric under varying data transfer bandwidths. Compression methods are ranked from left (compression ratio prioritized) to right ((de)compression speed prioritized), reflecting optimal choices depending on network or I/O constraints.

To illustrate the practical implications of B_max_, we computed and ranked compression algorithms under varying network transfer bandwidths (Fig 5). The results highlight the dynamic nature of optimal compressor selection based on bandwidth availability:

- Under low transfer bandwidth conditions, compression algorithms with higher compression ratios—such as Pcodec, bzip2, and LZMA with high compression levels—perform better, as the reduced data volume offsets their slower processing speeds.
- At high bandwidth levels (e.g., in high-speed server clusters with dedicated interconnects), fast compression and decompression algorithms like Pcodec, Blosc-LZ4, or LZ4 with low compression levels are favored. In these cases, data transfer is no longer the limiting factor, so maximizing processing speed becomes the priority.
- At mid-range bandwidths (e.g., 1–10 Gbps, typical of modern Ethernet infrastructure speed standardized in 1999 and 2006 [59,60]), balanced strategies are preferred. Compression methods that offer consistent, moderate performance across all metrics are most advantageous in these commonly encountered environments.

Compression algorithms are ordered from left to right based on shifting emphasis from compression ratio (low-bandwidth scenarios) to (de)compression speed (high-bandwidth scenarios), reflecting their optimal applicability under different network constraints.

For systems subject to fluctuating network conditions or requiring broad interoperability, it is essential to select a compressor that performs reliably across diverse bandwidth scenarios. To address this, we evaluated the worst-case performance of each compression algorithm across multiple transfer bandwidths. Pcodec showed the most balanced overall performance, followed by Blosc-zstd and zstd (Table 4). Their strong performance stems from the use of tANS-based entropy coding, which enables efficient fractional-bit encoding and near-optimal compression ratios with fast table-based decoding [61,62]. Zstd combines dictionary compression with tANS to balance speed and compression efficiency [36], Blosc improves zstd throughput via optimized CPU cache utilization [44], and Pcodec further enhances performance through numerical-data-specific preprocessing, including latent variable decomposition and delta encoding [42].

**Table 4:** All-rounder compression options based on the throughput-based metric.

Ranking	1^st^	2^nd^	3^rd^
All-rounder compression option	Pcodec (M)	Blosc-zstd (M)	zstd (M)

These compression options strike an effective balance between high compression ratios and fast processing speeds, making them suitable for a wide array of applications, including cloud-based HT reconstruction, streaming visualization, and AI-powered scientific computing.

## 4. Discussion

In this study, we systematically evaluated data compression strategies for HT imaging within the OME-Zarr framework, aiming to optimize storage efficiency and facilitate scalable data sharing. As HT imaging continues to produce increasingly large and high-resolution datasets, effective compression has become essential—not only for minimizing storage footprints and data transfer costs but also for enabling seamless accessibility in collaborative, cloud-based research environments.

Given the chunked and cloud-native nature of OME-Zarr, we investigated how different combinations of preprocessing filters and compression algorithms influence both storage and computational performance in realistic HT workflows. Our benchmark results demonstrate that the optimal choice of compression strategy depends heavily on the available data transfer bandwidth. In low-bandwidth scenarios, algorithms with higher compression ratios reduce transmission time despite slower processing speeds. Conversely, in high-bandwidth settings, fast compression methods are more effective due to reduced computational bottlenecks. For bandwidth conditions typical of modern Ethernet environments (1–10 Gbps), balanced options such as Pcodec (Medium), Blosc-zstd (Medium), and zstd (Medium) provide robust performance across metrics, making them suitable for general-purpose use.

A key consideration is the transition to OME-Zarr v0.5 (Zarr v3), which introduces optional sharding. Sharding reduces file count and metadata overhead, improving cloud performance, but may limit parallelism depending on shard size. Our benchmarks, based on v0.4 (Zarr v2) with per-chunk files, may see absolute throughput shifts under sharding, though relative codec rankings should remain consistent. Future work should validate the throughput metric under v0.5 sharded layouts.

We focused on codecs commonly deployed via Numcodecs in OME-Zarr to maximize reproducibility across current HT pipelines. SZ, MGARD, and FPZIP are established error-bounded compressors in scientific HPC; while not included in our main matrix, they merit evaluation under RI-aware error budgets (e.g., |*Δn*| ≤ 10^−4^) and downstream tasks. Future studies could consider a targeted extension comparing these tools against the tested compression options on organoid, embryo, and tensor datasets.

To illustrate the practical utility of the proposed metric, we consider a representative scenario in which the Embryo dataset is transferred over a network with an upload bandwidth of 100 MiB/s, a value typical of institutional internet connections. Without compression, the effective transfer rate is limited by the raw upload bandwidth. In contrast, applying Pcodec at a medium compression level yields a maximum achievable throughput of approximately 210 MiB/s, corresponding to an improvement of up to 2.1× in effective data transfer (see S1 Table for calculation details). This example demonstrates how the benchmark results can directly inform compression strategy selection to accelerate data sharing in real-world HT workflows.

Although the benchmarks were conducted on a single workstation, the results are designed to generalize to distributed computing environments. Such systems typically consist of multiple processing nodes interconnected by network infrastructure. The proposed throughput-based metric explicitly incorporates network transfer bandwidth, enabling per-node compression and decompression performance to be combined with measured or estimated network speeds to predict system-level throughput. Furthermore, the chunk-level parallelism inherent to the OME-Zarr format supports scalable deployment, as independent chunks can be processed concurrently across distributed nodes. We acknowledge, however, that real-world distributed environments may introduce additional factors not captured in our benchmarks, including network latency variability, concurrent access contention, and cloud storage I/O fluctuations. Future work will involve validating these predictions through benchmarks conducted on actual distributed and cloud-based infrastructures.

Also, future work should expand upon this benchmarking by exploring additional compression methods and filtering strategies specialized for specific hardware or scenarios. For example, NVIDIA offers GPU-accelerated compression algorithms [63]. They are not thoroughly tested in this work because the use case is not suitable in the cloud-based HT reconstruction and analysis scenario. When applying GPU-based compressors, data travel between memory and GPU back and forth, resulting in a memory bus bottleneck (S2 Fig). Another important direction is optimizing chunk size and shape, as these parameters significantly impact read/write performance and access latency in parallel or cloud-based data processing workflows [64].

Beyond performance optimization, our findings highlight the broader advantages of adopting the Zarr and OME-Zarr ecosystem over other prevalent formats, such as TIFF and HDF5. Moore et al. benchmarked bioimage data access latency across local, HTTP, and cloud storage environments [25]. While Zarr and HDF5 showed similar performance on local storage and both outperformed TIFF, Zarr demonstrated at least an order-of-magnitude faster access than HDF5 in cloud settings, enabled by direct chunk-level access. These next-generation formats offer a simplified design, excellent support for distributed access, and compatibility with cloud infrastructure. Despite being relatively new, Zarr has already been implemented across multiple programming languages and platforms, and its development continues through active contributions from a wide community of researchers, developers, and industry stakeholders.

The HT imaging community is well-positioned to play a proactive role in shaping the future of Zarr-based data management. By contributing domain-specific use cases, feature requests, and performance insights, HT researchers can help tailor the evolution of OME-Zarr to meet the unique demands of high-throughput, multidimensional imaging.

In conclusion, this study provides a comprehensive baseline for compression strategy selection within the OME-Zarr framework and supports the ongoing development of FAIR, high-performance data infrastructure for HT. Through collaborative and open innovation, the community can continue to enhance data sharing, analysis, and archiving practices across the biomedical imaging field.

## 5. Code availability

The complete benchmarking workflow, including scripts, configuration files, and detailed command-line instructions, is available at the following GitHub repository (https://github.com/Biomedical-optics-lab-KAIST/ht-data-ome-zarr-benchmark or https://github.com/ehgus/ht-data-ome-zarr-benchmark).

## Supporting information

S1 Fig Visual assessment of information loss bit-rounding processing. The slices of the original and filtered HT images are displayed with their difference images. The difference images are visualized in a scaled range with the maximum absolute value.
(TIF)

S2 Fig Benchmark results of zlib and LZ4 on CPU and GPU. The left panels show compression with no filter (blue), byte shuffle (green), bit round (red), and combined bit round + byte shuffle (orange). The right panels show compression and decompression bandwidth in different compression levels: low, medium, and high.
(TIF)

S1 Table Full benchmark results. Compression metric values—compression ratio, compression speed, decompression speed—are displayed for each combination of datasets, compressor options, and filter options.
(CSV)
