# Supplementary figures and images for: Compression benchmarking of holotomography data using OME-Zarr format
Source: PLoS One. 2026 Jul 8;21(7):e0351560. doi: 10.1371/journal.pone.0351560 (PMC13345466; doi:10.1371/journal.pone.0351560)

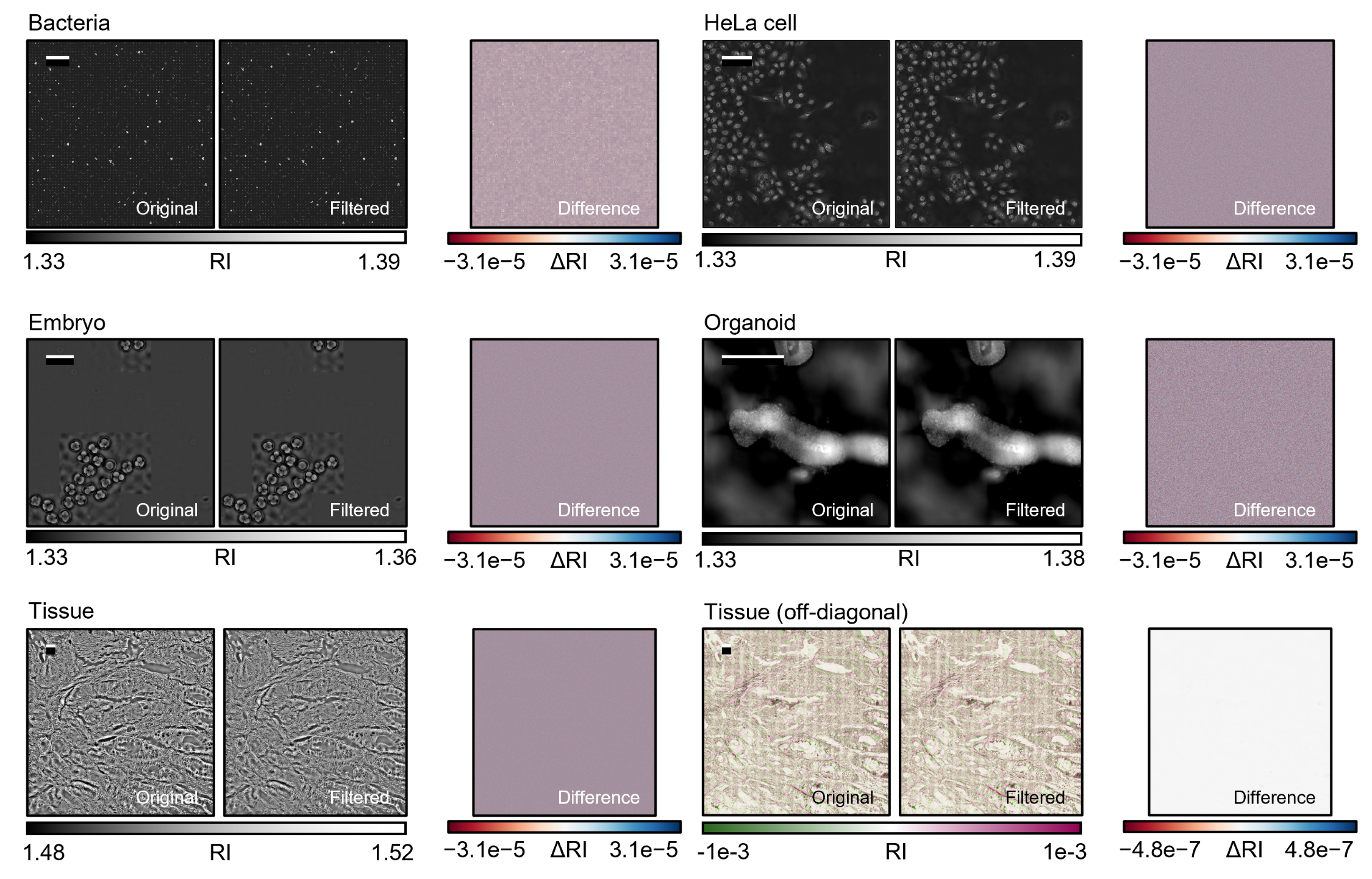

Supplement: S1 Fig — The slices of the original and filtered HT images are displayed with their difference images. The difference images are visualized in a scaled range with the maximum absolute value. (TIF) [file pone.0351560.s001.tif]

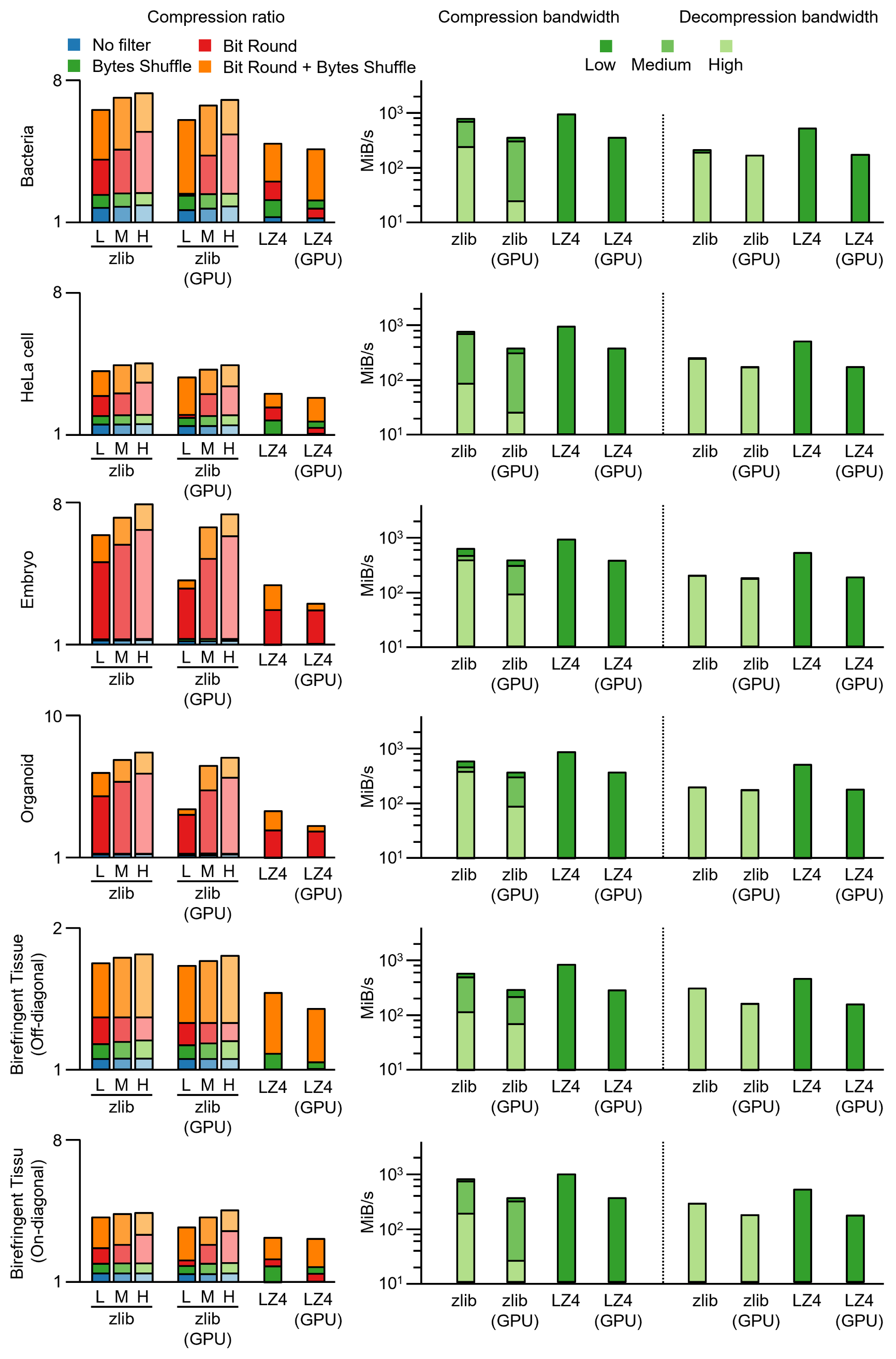

Supplement: S2 Fig — The left panels show compression with no filter (blue), byte shuffle (green), bit round (red), and combined bit round + byte shuffle (orange). The right panels show compression and decompression bandwidth in different compression levels: low, medium, and high. (TIF) [file pone.0351560.s002.tif]
