# Supplementary material for: Compression benchmarking of holotomography data using OME-Zarr format
Source: PLoS One. 2026 Jul 8;21(7):e0351560. doi: 10.1371/journal.pone.0351560 (PMC13345466; doi:10.1371/journal.pone.0351560)
Supplement: Attachment — Submitted filename: plos-one-response-rebuttal-letter_v2.1.docx [file pone.0351560.s005.docx]

Reviewer #1: The paper presents a sound and thorough analysis of compression algorithms applied to HT data. The manuscript is well-structured, clearly written, and easy to follow. The experimental evaluation is solid and well designed, covering both compression and decompression performance effectively.

However, the statement that the sampling strategy “preserves data diversity representative of real-world scenarios” would benefit from additional support or justification — for instance, through a quantitative assessment of data diversity or a more detailed explanation of how representativeness was ensured during sampling.

We appreciate the reviewer’s constructive feedback regarding the justification of representativeness in our sampling strategy.

In the revised Methods section, we have clarified that our random sampling procedure draws chunks from diverse spatial locations across the full 3D volume, rather than from a contiguous region, thereby capturing a range of local structural characteristics (e.g., cellular regions, background-dominant areas, and heterogeneous interfaces) that are typically encountered in real-world holotomography datasets.

While we acknowledge that this sampling approach does not exhaustively enumerate all possible data patterns within the full volume, its goal is not statistical completeness but practical representativeness for compression benchmarking. By uniformly sampling from spatially separated locations, the selected chunks span variations in signal sparsity, structural complexity, and refractive-index distribution that are most relevant to evaluating compression performance.

We have revised the manuscript text to explicitly state this scope and limitation, and to frame representativeness in an operational sense—namely, capturing the diversity of structural features typically encountered during data storage and transmission workflows—rather than as a claim of full volumetric coverage.

Additionally, it could be helpful to visually highlight the best-performing algorithm(s) in the figures, which would make the comparison clearer and improve the overall readability of the experimental results.

We appreciate the reviewer’s helpful suggestion.

To improve clarity and readability, we have visually highlighted the best-performing algorithm(s) in Figure 5 by enhancing their graphical emphasis (e.g., bold outlines and distinct visual markers), allowing readers to more easily identify the top-performing methods at a glance. This modification improves the interpretability of the comparative results without altering the underlying data or analysis.

Reviewer #2: The manuscript titled “Compression benchmarking of holotomography data using OME-Zarr format” presents a benchmarking of data compression strategies for holotomography data stored in the OME-Zarr format, using six representative datasets from five biological samples, where 4 kinds of utilization of preprocessing filters and 13 compression algorithms across multiple compression levels are tested. The work is systematic and comprehensive, and it is worth of publishing after some essential improvements and revisions. Here are the comments:

\# Major

1. It is not clear what are the key insights. It would be better to clarify them at the beginning of the manuscript. It is also recommended to state how this benchmark is used to guide and improve the workflow process or corresponding compression methods, to enhance the impact of the manuscript.

We appreciate the reviewer’s valuable suggestion.

In response, we have revised both the Abstract and Introduction to clearly articulate the key insights of this study at the outset. Specifically, we now emphasize that the optimal compression strategy for holotomography data is determined by the available network bandwidth, and that Pcodec consistently emerges as the most balanced, all-around performer across a wide range of practical conditions.

In addition, we explicitly describe how this benchmark can be used to guide real-world workflow decisions, such as selecting appropriate compression methods for data storage, remote transfer, and high-throughput imaging pipelines. By framing the benchmark as a decision-support tool rather than a purely comparative evaluation, we clarify its practical value in improving end-to-end holotomography data workflows.

2. The manuscript reads like all tests were performed on one workstation, instead of a real distributed system. It is unclear whether the test results can be reliably applied in the real-world scenario; clarifying possible limitations may help.

We appreciate the reviewer’s thoughtful comment regarding the applicability of our results to real-world distributed systems.

As clarified in the revised Discussion section, although all benchmarks were conducted on a single workstation, our analysis is formulated around a throughput-based metric that can be directly combined with the effective internal bandwidth of a distributed system to estimate system-level data transfer performance. This abstraction allows the benchmarking results to inform compression choices beyond the specific hardware used in our experiments.

At the same time, we explicitly acknowledge the limitations of this approach. Real-world distributed environments may introduce additional factors—such as network latency, concurrency, I/O contention, and system heterogeneity—that are not captured in a single-node benchmark. These factors are now discussed as limitations, and we frame our results as providing practical guidance rather than exact performance prediction for distributed deployments.

This clarification positions the benchmark as a scalable reference that remains informative under realistic system constraints, while transparently defining its scope.

3. Why some datasets were collected by a customized system, while others are not. It is recommended to clarify the reason.

We appreciate the reviewer’s request for clarification regarding the use of both commercial and custom-built imaging systems across the datasets.

As clarified in the revised manuscript, custom-built systems were employed only when commercial holotomography platforms could not satisfy specific experimental requirements. In particular, for embryo imaging, a custom setup was necessary to achieve embryo-compatible acquisition conditions with reduced phototoxicity, as previously reported. For birefringent tissue imaging, a fully custom-built polarization-sensitive holotomography system was required, since current commercial HT systems do not support birefringence measurements.

In contrast, datasets that did not impose such specialized constraints were acquired using standard commercial systems. This design serves a dual purpose. First, by including benchmarks obtained directly from existing commercial platforms, the study provides immediately actionable guidance for researchers seeking to improve data storage and transfer workflows in current practice. Second, by incorporating datasets acquired using custom-built systems, the benchmark is intentionally extended to emerging and non-commercial HT modalities, offering forward-looking guidelines for future method development and system design.

We have revised the manuscript to explicitly state these criteria, emphasizing that the choice of imaging system was driven by measurement requirements and the intended scope of applicability, rather than methodological inconsistency.

4. It would be better to select a specific application to verify whether the benchmark can assist in promoting the workflow.

We appreciate the reviewer’s suggestion to validate the benchmark through a concrete application scenario.

To address this point, we have added a specific, workflow-oriented use case in the Discussion section. Using the Embryo dataset as an example, we demonstrate how the benchmark can directly inform compression choices in a realistic data-transfer setting. Specifically, when transferring the dataset over a typical desktop connection with an upload bandwidth of approximately 100 MiB/s, selecting Pcodec at a medium compression level results in an effective transfer speed improvement of up to 2.1× compared to uncompressed data.

Importantly, this example illustrates how the benchmark functions as a decision-support tool for optimizing data transfer workflows under practical network constraints. To ensure transparency and reproducibility, we have also added a Supplementary Table that reports the detailed benchmark results for all samples and compression configurations used in this analysis.

5. It is highly recommended to provide reasons on why combinations like “Pcodec (M)” and “blosc-zstd (M)” can reach the highest performance in the proposed metric.

We appreciate the reviewer’s insightful question regarding the underlying reasons why combinations such as Pcodec (M) and blosc-zstd (M) achieve the highest performance under the proposed metric.

As clarified in the revised Results section, both Pcodec and zstd employ tANS-based entropy coding, which provides near-optimal compression efficiency while maintaining high processing speed. This balance is particularly advantageous for our throughput-based metric, which jointly reflects compression ratio and computational cost.

In addition, Pcodec further improves performance through optimized data preprocessing steps that enhance entropy coding efficiency for holotomography data, while Blosc augments zstd by maximizing CPU cache utilization and memory access efficiency during chunk-wise compression. Together, these design features explain why medium-level configurations of these methods consistently outperform alternatives in the proposed benchmark.

\# Minor

1. It would be better to introduce some basic concepts, such as compression ratio and the relationship between the ratio and speed performance.

We have added brief explanations of the trade-off between compression ratio and processing speed in the Methods and Results sections.

2. It is recommended to state the quantitative values to describe the “Level” in Table 2.

We have revised the Table 2 caption to clarify that the numeric values indicate algorithm-specific compression levels corresponding to Low, Medium, and High settings.

3. It is recommended to avoid such a big blank on Page 10.

We have adjusted the figure placement to remove the blank space on Page 10. Thank you for pointing this out.

4. It would be better to mention some quantitative disadvantages of TIFF and HDF5 compared with Zarr.

We have expanded the Discussion to address the quantitative disadvantages of TIFF and HDF5. Moore et al. conducted a bioimage latency benchmark comparing these formats [1]. On local storage, Zarr and HDF5 showed similar performance, both outperforming TIFF. On cloud storage, Zarr was an order of magnitude faster than HDF5. We have added this reference and summarized these findings in the revised manuscript.

Reviewer #3: The manuscript “Compression benchmarking of holotomography data using OME-Zarr format” focuses on the interesting topic of efficient data compression for very large 3D imaging database, namely Holotomography (HT).

The proposed investigation considers the OME-Zarr format, discussing how the HT data can be managed within this this compressed data storage format. As for any compression strategy, unlike preliminary preprocessing steps, the data encoding strategy is the core to achieve advanced data management in terms of compression ratio, compression and decompression bandwidth, quality of the recovered data (i.e. decoded data).

The authors provide a comprehensive analysis by including several datasets acquired in different HT systems and a systematic analysis of the widely used compression algorithms available within the OME-Zarr format.

My general opinion is that the manuscript is well-written and suitable for the publication in PLOS ONE after addressing the following major concerns

1) The authors discussed on the use of OME-Zarr format for HT data compression but without considering other potential alternative. Please provide details and discussion about other solutions even including the expected differences with respect the OME-Zarr format coding in terms of the overall efficiency.

Thank you for raising this important point regarding alternative data formats.

In the revised Discussion, we have expanded the comparison of OME-Zarr with other widely used bioimaging data formats, specifically TIFF and HDF5, which represent common baselines for both legacy workflows and high-performance scientific data storage. We discuss the expected differences in overall efficiency in terms of chunked access, parallel I/O, compression flexibility, and suitability for cloud and distributed environments.

In particular, we reference the bioimage latency benchmark study by Moore et al. [1], which systematically compared TIFF, HDF5, and Zarr formats and demonstrated that Zarr provides superior performance for both local random access and cloud-based data access, largely due to its native chunking and object-storage-friendly design. These results are consistent with the workflow requirements of large-scale holotomography data and support our choice of OME-Zarr as the primary storage and compression framework.

We note that other formats (e.g., HDF5-based solutions) can offer efficient performance in specific local or monolithic storage scenarios; however, their scalability and flexibility are more limited in distributed and cloud-oriented workflows. We have clarified these trade-offs in the manuscript to better contextualize the advantages and scope of OME-Zarr in our benchmarking framework.

2) Since HT devices considered in this study are commercialized by part of some authors (if I understood correctly), what is the compression data pipeline used in the past for HT data management? Please add these information underlining the advantages using the current OME-Zarr format

We appreciate the reviewer’s thoughtful question regarding the data compression pipeline historically used for HT data management, particularly in the context of commercially available systems.

We have clarified in the revised Introduction the general characteristics of prior HT data management practices. In existing workflows, HT data have typically been stored using basic preprocessing and conventional lossless compression schemes, without systematic optimization of entropy coding, chunking strategies, or format-level support for scalable access and distributed workflows.

Importantly, these legacy approaches were largely driven by hardware and software constraints at the time of system deployment, rather than by a comprehensive evaluation of compression efficiency, throughput, or workflow scalability. As a result, compression strategies for HT data have not been rigorously benchmarked or standardized across the field.

We have added this context to motivate the present study and to highlight the advantages of adopting the OME-Zarr format, which enables chunk-wise compression, flexible integration of modern entropy-based codecs, and efficient access in local, distributed, and cloud-based environments. This positioning clarifies that our work is not a replacement of a known optimal commercial pipeline, but rather a systematic, format-agnostic benchmarking effort aimed at establishing transparent and reproducible guidelines for HT data management.

3) The study reports on some performance metric including compression ratio, compression bandwidth and decompression bandwidth. Moreover the authors propose a new throughput-based evaluation metric for generalizing the quantification of the performance of a compression strategy in realistic scenarios. Although this analysis is very interesting, I believe there is a lack of a more direct metric to quantify the information loss caused by data compression. Basically, for a general reader, It would be really useful to "see" some HT images going through the preprocessing/compression/decompression stages. Basically, I suggest to include a figure reporting an uncompressed HT image (as it is obtained from the imaging system) and the same after using the decompression from the OME-Zarr format. Possibly, some similarity metrics between these images (e.g. SSIM, MSE) could help to understand the information loss of the data depending of the selected compression strategy

We appreciate the reviewer’s thoughtful suggestion to include a more direct and intuitive assessment of potential information loss, particularly through visual comparison of HT images before and after compression.

We would first like to clarify an important point: all compression algorithms evaluated in this study are strictly lossless, meaning that the decompressed data are mathematically identical to the original input data, with no information loss introduced by the compression stage itself. As such, similarity metrics such as SSIM or MSE between uncompressed and decompressed images would be identically zero and therefore uninformative for evaluating the compression algorithms.

However, the reviewer correctly points out that the bit-rounding filter, which is an optional preprocessing step applied prior to compression, is a lossy operation that reduces floating-point precision. To address this concern, we have expanded the Methods section to clearly distinguish between lossless compression and lossy preprocessing, and we have added a new Supplementary Figure S2. This figure presents representative HT images in three stages: the original image, the image after bit-rounding, and the corresponding difference image.

In our implementation, the bit-rounding filter was configured to preserve four significant digits, ensuring that any numerical differences remain within a user-defined precision threshold. The difference images in Supplementary Figure S2 visually confirm that the induced changes are minimal and well controlled. This addition allows readers to directly assess the magnitude and nature of precision loss associated with preprocessing, while reaffirming that the compression stage itself remains fully lossless.

4) Even if the authors claim that “Although prior studies have reported compression benchmarks in other imaging contexts [29-31], a comprehensive evaluation tailored to HT imaging has not been performed”, some studies are reported for some 3D imaging modalities also including HT. Please see and comment about the following papers

• B. Subramanian et al., “On a hybrid lossless compression technique for three-dimensional medical images,” J Appl Clin Med Phys 2021;22(8):191–203.

• O.H. Nagoor et al., “Sampling strategies for learning-based 3D medical image compression,” Machine Learning with Applications 8 (2022) 100273.

• H.M. Luu et al., “Efficiently compressing 3D medical images for teleinterventions via CNNs and anisotropic diffusion,” Med Phys 2021 Jun;48(6):2877-2890.

• P. Memmolo et al., “Loss Minimized Data Reduction in Single-Cell Tomographic Phase Microscopy Using 3D Zernike Descriptors,” Intell. Comput. 2023;2:Article 0010.

We thank the reviewer for bringing these valuable references to our attention. We have reviewed each of the suggested papers and would like to clarify how our work differs in scope and objective.

The cited studies primarily focus on developing or optimizing specific compression methods for 3D medical or tomographic imaging. For example, Subramanian et al. propose a hybrid lossless compression technique for 3D medical images; Nagoor et al. and Luu et al. investigate learning-based or CNN-driven compression strategies; and Memmolo et al. introduce a domain-specific data reduction approach for tomographic phase microscopy using 3D Zernike descriptors. These works provide valuable algorithmic advances and demonstrate the effectiveness of particular methods within their respective contexts.

In contrast, the primary goal of our study is not to propose a new compression algorithm, but to establish a systematic, application-driven benchmarking framework tailored to holotomography (HT) data. Specifically, we evaluate a broad range of existing, widely used compression algorithms and configurations under realistic workflow constraints, including storage, data transfer, and network bandwidth limitations. Our proposed throughput-based metric is designed to support practical decision-making across diverse deployment scenarios, rather than to optimize a single compression method.

While some of the cited works include tomographic or phase-based imaging modalities, none, to our knowledge, provide a comprehensive, bandwidth-aware benchmarking study focused on HT data workflows, nor do they evaluate compression strategies across both commercial and emerging HT systems within a unified framework.

We have revised the manuscript to explicitly acknowledge these related studies and to clarify the distinct contribution of our work as a benchmarking and standardization-oriented reference, complementing prior algorithm-centric research rather than replacing it.

References

1.	Moore J, Allan C, Besson S, Burel J-M, Diel E, Gault D, et al. OME-NGFF: a next-generation file format for expanding bioimaging data-access strategies. Nat Methods. 2021;18: 1496–1498. doi:10.1038/s41592-021-01326-w
