# Supplementary material for: Compression benchmarking of holotomography data using OME-Zarr format
Source: PLoS One. 2026 Jul 8;21(7):e0351560. doi: 10.1371/journal.pone.0351560 (PMC13345466; doi:10.1371/journal.pone.0351560)
Supplement: Peer review history

# Peer review: Decision Letter 0

**DOI:** 10.1371/journal.pone.0351560.r001

**Authors:** Francesco Bardozzo

10 Nov 2025

PONE-D-25-48731

Compression benchmarking of holotomography data using OME-Zarr format

PLOS ONE

Dear Dr. Lee,

Thank you for submitting your manuscript to PLOS ONE. After careful consideration, we feel that it has merit but does not fully meet PLOS ONE’s publication criteria as it currently stands. Therefore, we invite you to submit a revised version of the manuscript that addresses the points raised during the review process.

Please submit your revised manuscript by Dec 25 2025 11:59PM. If you will need more time than this to complete your revisions, please reply to this message or contact the journal office at plosone@plos.org. When you're ready to submit your revision, log on to https://www.editorialmanager.com/pone/ and select the 'Submissions Needing Revision' folder to locate your manuscript file.

Please include the following items when submitting your revised manuscript:

- A rebuttal letter that responds to each point raised by the academic editor and reviewer(s). You should upload this letter as a separate file labeled 'Response to Reviewers'.
- A marked-up copy of your manuscript that highlights changes made to the original version. You should upload this as a separate file labeled 'Revised Manuscript with Track Changes'.
- An unmarked version of your revised paper without tracked changes. You should upload this as a separate file labeled 'Manuscript'.

If you would like to make changes to your financial disclosure, please include your updated statement in your cover letter. Guidelines for resubmitting your figure files are available below the reviewer comments at the end of this letter.

If applicable, we recommend that you deposit your laboratory protocols in protocols.io to enhance the reproducibility of your results. Protocols.io assigns your protocol its own identifier (DOI) so that it can be cited independently in the future. For instructions see: https://journals.plos.org/plosone/s/submission-guidelines#loc-laboratory-protocols. Additionally, PLOS ONE offers an option for publishing peer-reviewed Lab Protocol articles, which describe protocols hosted on protocols.io. Read more information on sharing protocols at https://plos.org/protocols?utm_medium=editorial-email&utm_source=authorletters&utm_campaign=protocols.

We look forward to receiving your revised manuscript.

Kind regards,

Francesco Bardozzo

Academic Editor

PLOS ONE

Journal Requirements:

When submitting your revision, we need you to address these additional requirements.

1. Please ensure that your manuscript meets PLOS ONE's style requirements, including those for file naming. The PLOS ONE style templates can be found at https://journals.plos.org/plosone/s/file?id=wjVg/PLOSOne_formatting_sample_main_body.pdf and https://journals.plos.org/plosone/s/file?id=ba62/PLOSOne_formatting_sample_title_authors_affiliations.pdf

2. Your ethics statement should only appear in the Methods section of your manuscript. If your ethics statement is written in any section besides the Methods, please delete it from any other section.

3. We noted in your submission details that a portion of your manuscript may have been presented or published elsewhere. The benchmark datasets of bacteria, HeLa cells, and organoids were originally reported in previous publications. However, the reuse of these data does not constitute dual publication. The earlier work focused on biological insights, whereas the present study addresses data compression, which is an entirely different research objective.

Please clarify whether this [conference proceeding or publication] was peer-reviewed and formally published. If this work was previously peer-reviewed and published, in the cover letter please provide the reason that this work does not constitute dual publication and should be included in the current manuscript.

4. We note that the grant information you provided in the ‘Funding Information’ and ‘Financial Disclosure’ sections do not match.

When you resubmit, please ensure that you provide the correct grant numbers for the awards you received for your study in the ‘Funding Information’ section.

5. Thank you for stating the following financial disclosure:

This work was supported by National Research Foundation of Korea grant funded by the Korea government (MSIT) (RS-2024-00442348, 2022M3H4A1A02074314), Institute of Information & communications Technology Planning & Evaluation (IITP; 2021-0-00272) grant funded by the Korea government, Korea Institute for Advancement of Technology (KIAT) through the International Cooperative R&D program (P0028463), and the Korean Fund for Regenerative Medicine (KFRM) grant funded by the Korea government (the Ministry of Science and ICT and the Ministry of Health & Welfare) (21A0101L1-12).

Please state what role the funders took in the study. If the funders had no role, please state: "The funders had no role in study design, data collection and analysis, decision to publish, or preparation of the manuscript."

If this statement is not correct you must amend it as needed.

Please include this amended Role of Funder statement in your cover letter; we will change the online submission form on your behalf.

6. Thank you for stating the following in the Acknowledgments Section of your manuscript:

This work was supported by National Research Foundation of Korea grant funded by the Korea government (MSIT) (RS-2024-00442348, 2022M3H4A1A02074314), Institute of Information & communications Technology Planning & Evaluation (IITP; 2021-0-00272) grant funded by the Korea government, Korea Institute for Advancement of Technology (KIAT) through the International Cooperative R&D program (P0028463), and the Korean Fund for Regenerative Medicine (KFRM) grant funded by the Korea government (the Ministry of Science and ICT and the Ministry of Health & Welfare) (21A0101L1-12).

We note that you have provided funding information that is not currently declared in your Funding Statement. However, funding information should not appear in the Acknowledgments section or other areas of your manuscript. We will only publish funding information present in the Funding Statement section of the online submission form.

Please remove any funding-related text from the manuscript and let us know how you would like to update your Funding Statement. Currently, your Funding Statement reads as follows:

This work was supported by National Research Foundation of Korea grant funded by the Korea government (MSIT) (RS-2024-00442348, 2022M3H4A1A02074314), Institute of Information & communications Technology Planning & Evaluation (IITP; 2021-0-00272) grant funded by the Korea government, Korea Institute for Advancement of Technology (KIAT) through the International Cooperative R&D program (P0028463), and the Korean Fund for Regenerative Medicine (KFRM) grant funded by the Korea government (the Ministry of Science and ICT and the Ministry of Health & Welfare) (21A0101L1-12).

Please include your amended statements within your cover letter; we will change the online submission form on your behalf.

7. Please amend your list of authors on the manuscript to ensure that each author is linked to an affiliation. Authors’ affiliations should reflect the institution where the work was done (if authors moved subsequently, you can also list the new affiliation stating “current affiliation:….” as necessary).

8. Please include captions for your Supporting Information files at the end of your manuscript, and update any in-text citations to match accordingly. Please see our Supporting Information guidelines for more information: http://journals.plos.org/plosone/s/supporting-information.

9. If the reviewer comments include a recommendation to cite specific previously published works, please review and evaluate these publications to determine whether they are relevant and should be cited. There is no requirement to cite these works unless the editor has indicated otherwise.

Additional Editor Comments:

I suggest following the reviewer comments.

[Note: HTML markup is below. Please do not edit.]

Reviewers' comments:

Reviewer's Responses to Questions

**Comments to the Author**

1. Is the manuscript technically sound, and do the data support the conclusions?

The manuscript must describe a technically sound piece of scientific research with data that supports the conclusions. Experiments must have been conducted rigorously, with appropriate controls, replication, and sample sizes. The conclusions must be drawn appropriately based on the data presented.

Reviewer #1: Yes

Reviewer #2: Yes

Reviewer #3: Partly

**********

2. Has the statistical analysis been performed appropriately and rigorously?

Reviewer #1: Yes

Reviewer #2: Yes

Reviewer #3: Yes

**********

3. Have the authors made all data underlying the findings in their manuscript fully available?

The PLOS Data policy requires authors to make all data underlying the findings described in their manuscript fully available without restriction, with rare exception (please refer to the Data Availability Statement in the manuscript PDF file). The data should be provided as part of the manuscript or its supporting information, or deposited to a public repository. For example, in addition to summary statistics, the data points behind means, medians and variance measures should be available. If there are restrictions on publicly sharing data—e.g. participant privacy or use of data from a third party—those must be specified.

Reviewer #1: Yes

Reviewer #2: Yes

Reviewer #3: Yes

**********

4. Is the manuscript presented in an intelligible fashion and written in standard English?

PLOS ONE does not copyedit accepted manuscripts, so the language in submitted articles must be clear, correct, and unambiguous. Any typographical or grammatical errors should be corrected at revision, so please note any specific errors here.

Reviewer #1: Yes

Reviewer #2: Yes

Reviewer #3: Yes

**********

5. Review Comments to the Author

Please use the space provided to explain your answers to the questions above. You may also include additional comments for the author, including concerns about dual publication, research ethics, or publication ethics. (Please upload your review as an attachment if it exceeds 20,000 characters)

Reviewer #1: The paper presents a sound and thorough analysis of compression algorithms applied to HT data. The manuscript is well-structured, clearly written, and easy to follow. The experimental evaluation is solid and well designed, covering both compression and decompression performance effectively.

However, the statement that the sampling strategy “preserves data diversity representative of real-world scenarios” would benefit from additional support or justification — for instance, through a quantitative assessment of data diversity or a more detailed explanation of how representativeness was ensured during sampling.

Additionally, it could be helpful to visually highlight the best-performing algorithm(s) in the figures, which would make the comparison clearer and improve the overall readability of the experimental results.

Reviewer #2: The manuscript titled “Compression benchmarking of holotomography data using OME-Zarr format” presents a benchmarking of data compression strategies for holotomography data stored in the OME-Zarr format, using six representative datasets from five biological samples, where 4 kinds of utilization of preprocessing filters and 13 compression algorithms across multiple compression levels are tested. The work is systematic and comprehensive, and it is worth of publishing after some essential improvements and revisions. Here are the comments:

# Major

1. It is not clear what are the key insights. It would be better to clarify them at the beginning of the manuscript. It is also recommended to state how this benchmark is used to guide and improve the workflow process or corresponding compression methods, to enhance the impact of the manuscript.

2. The manuscript reads like all tests were performed on one workstation, instead of a real distributed system. It is unclear whether the test results can be reliably applied in the real-world scenario; clarifying possible limitations may help.

3. Why some datasets were collected by a customized system while others are not. It is recommended to clarify the reason.

4. It would be better to select a specific application to verify whether the benchmark can assist in promoting the workflow.

5. It is highly recommended to provide reasons on why combinations like “Pcodec (M)” and “blosc-zstd (M)” can reach the highest performance in the proposed metric.

# Minor

1. It would be better to introduce some basic concepts, such as compression ratio and the relationship between the ratio and speed performance.

2. It is recommended to state the quantitative values to describe the “Level” in Table 2.

3. It is recommended to avoid such a big blank on Page 10.

4. It would be better to mention some quantitative disadvantages of TIFF and HDF5 compared with Zarr.

Reviewer #3: The manuscript “Compression benchmarking of holotomography data using OME-Zarr format” focuses on the interesting topic of efficient data compression for very large 3D imaging database, namely Holotomography (HT).

The proposed investigation considers the OME-Zarr format, discussing how the HT data can be managed within this this compressed data storage format. As for any compression strategy, unlike preliminary preprocessing steps, the data encoding strategy is the core to achieve advanced data management in terms of compression ratio, compression and decompression bandwidth, quality of the recovered data (i.e. decoded data).

The authors provide a comprehensive analysis by including several datasets acquired in different HT systems and a systematic analysis of the widely used compression algorithms available within the OME-Zarr format.

My general opinion is that the manuscript is well-written and suitable for the publication in PLOS ONE after addressing the following major concerns

1) The authors discussed on the use of OME-Zarr format for HT data compression but without considering other potential alternative. Please provide details and discussion about other solutions even including the expected differences with respect the OME-Zarr format coding in terms of the overall efficiency.

2) Since HT devices considered in this study are commercialized by part of some authors (if I understood correctly), what is the compression data pipeline used in the past for HT data management? Please add these information underlining the advantages using the current OME-Zarr format.

3) The study reports on some performance metric including compression ratio, compression bandwidth and decompression bandwidth. Moreover the authors propose a new throughput-based evaluation metric for generalizing the quantification of the performance of a compression strategy in realistic scenarios. Although this analysis is very interesting, I believe there is a lack of a more direct metric to quantify the information loss caused by data compression. Basically, for a general reader, It would be really useful to "see" some HT images going through the preprocessing/compression/decompression stages. Basically, I suggest to include a figure reporting an uncompressed HT image (as it is obtained from the imaging system) and the same after using the decompression from the OME-Zarr format. Possibly, some similarity metrics between these images (e.g. SSIM, MSE) could help to understand the information loss of the data depending of the selected compression strategy.

4) Even if the authors claim that “Although prior studies have reported compression benchmarks in other imaging contexts [29-31], a comprehensive evaluation tailored to HT imaging has not been performed”, some studies are reported for some 3D imaging modalities also including HT. Please see and comment about the following papers

• B. Subramanian et al., “On a hybrid lossless compression technique for three-dimensional medical images,” J Appl Clin Med Phys 2021;22(8):191–203.

• O.H. Nagoor et al., “Sampling strategies for learning-based 3D medical image compression,” Machine Learning with Applications 8 (2022) 100273.

• H.M. Luu et al., “Efficiently compressing 3D medical images for teleinterventions via CNNs and anisotropic diffusion,” Med Phys 2021 Jun;48(6):2877-2890.

• P. Memmolo et al., “Loss Minimized Data Reduction in Single-Cell Tomographic Phase Microscopy Using 3D Zernike Descriptors,” Intell. Comput. 2023;2:Article 0010.

**********

6. PLOS authors have the option to publish the peer review history of their article (what does this mean?). If published, this will include your full peer review and any attached files.

If you choose “no”, your identity will remain anonymous but your review may still be made public.

**Do you want your identity to be public for this peer review?**  For information about this choice, including consent withdrawal, please see our Privacy Policy.

Reviewer #1: No

Reviewer #2: No

Reviewer #3: No

**********

[NOTE: If reviewer comments were submitted as an attachment file, they will be attached to this email and accessible via the submission site. Please log into your account, locate the manuscript record, and check for the action link "View Attachments". If this link does not appear, there are no attachment files.]

To ensure your figures meet our technical requirements, please review our figure guidelines: https://journals.plos.org/plosone/s/figures

You may also use PLOS’s free figure tool, NAAS, to help you prepare publication quality figures: https://journals.plos.org/plosone/s/figures#loc-tools-for-figure-preparation.

NAAS will assess whether your figures meet our technical requirements by comparing each figure against our figure specifications.

# Author comment: Author response to Decision Letter 1

**DOI:** 10.1371/journal.pone.0351560.r002

8 Jan 2026

We thank you for your time and consideration of our previous submission entitled “Compression benchmarking of holotomography data using the OME-Zarr storage format”. Thanks to the constructive comments by the reviewers, we have improved the quality of the manuscript.

In the attached rebuttal letter, we address the referee’s comments and a list of changes in our revised manuscript according to their reports. The original referee comments are provided in black, whereas our answers are given in blue. The appropriate changes made in the revised manuscript are highlighted. We have also taken the opportunity to make some additional changes to refine the manuscript's quality; these are also highlighted. Additionally, we have updated our financial disclosure to include an additional funding source (Samsung Research Funding Center of Samsung Electronics under Grant SRFC-IT1401-08) and our Competing Interests statement to note that Juyeon Park also holds financial interests in Tomocube Inc.

We believe that these modifications have strengthened the manuscript and hope that the revised manuscript is suitable for publication in PLOS One.

Attachment Submitted filename: plos-one-response-rebuttal-letter_v2.1.docx

# Peer review: Decision Letter 1

**DOI:** 10.1371/journal.pone.0351560.r003

**Authors:** Francesco Bardozzo, Carla Pegoraro

28 May 2026

Compression benchmarking of holotomography data using OME-Zarr format

PONE-D-25-48731R1

Dear Dr. Lee,

We’re pleased to inform you that your manuscript has been judged scientifically suitable for publication and will be formally accepted for publication once it meets all outstanding technical requirements.

Within one week, you’ll receive an e-mail detailing the required amendments. When these have been addressed, you’ll receive a formal acceptance letter and your manuscript will be scheduled for publication.

An invoice will be generated when your article is formally accepted. Please note, if your institution has a publishing partnership with PLOS and your article meets the relevant criteria, all or part of your publication costs will be covered. Please make sure your user information is up-to-date by logging into Editorial Manager at Editorial Manager® and clicking the ‘Update My Information' link at the top of the page. For questions related to billing, please contact billing support.

If your institution or institutions have a press office, please notify them about your upcoming paper to help maximize its impact. If they’ll be preparing press materials, please inform our press team as soon as possible -- no later than 48 hours after receiving the formal acceptance. Your manuscript will remain under strict press embargo until 2 pm Eastern Time on the date of publication. For more information, please contact onepress@plos.org.

Kind regards,

Carla Pegoraro

Staff Editor

PLOS One

Additional Editor Comments (optional):

Reviewers' comments:

Reviewer's Responses to Questions

**Comments to the Author**

1. If the authors have adequately addressed your comments raised in a previous round of review and you feel that this manuscript is now acceptable for publication, you may indicate that here to bypass the “Comments to the Author” section, enter your conflict of interest statement in the “Confidential to Editor” section, and submit your "Accept" recommendation.

Reviewer #1: All comments have been addressed

Reviewer #2: All comments have been addressed

**********

2. Is the manuscript technically sound, and do the data support the conclusions?

The manuscript must describe a technically sound piece of scientific research with data that supports the conclusions. Experiments must have been conducted rigorously, with appropriate controls, replication, and sample sizes. The conclusions must be drawn appropriately based on the data presented.

Reviewer #1: Yes

Reviewer #2: Yes

**********

3. Has the statistical analysis been performed appropriately and rigorously?

Reviewer #1: Yes

Reviewer #2: Yes

**********

4. Have the authors made all data underlying the findings in their manuscript fully available?

The PLOS Data policy requires authors to make all data underlying the findings described in their manuscript fully available without restriction, with rare exception (please refer to the Data Availability Statement in the manuscript PDF file). The data should be provided as part of the manuscript or its supporting information, or deposited to a public repository. For example, in addition to summary statistics, the data points behind means, medians and variance measures should be available. If there are restrictions on publicly sharing data—e.g. participant privacy or use of data from a third party—those must be specified.

Reviewer #1: Yes

Reviewer #2: Yes

**********

5. Is the manuscript presented in an intelligible fashion and written in standard English?

PLOS ONE does not copyedit accepted manuscripts, so the language in submitted articles must be clear, correct, and unambiguous. Any typographical or grammatical errors should be corrected at revision, so please note any specific errors here.

Reviewer #1: Yes

Reviewer #2: Yes

**********

6. Review Comments to the Author

Please use the space provided to explain your answers to the questions above. You may also include additional comments for the author, including concerns about dual publication, research ethics, or publication ethics. (Please upload your review as an attachment if it exceeds 20,000 characters)

Reviewer #1: (No Response)

Reviewer #2: We appreciate the authors' efforts in addressing all the comments and improving the manuscript, which is now more systematic and informative and ready to be accepted.

**********

7. PLOS authors have the option to publish the peer review history of their article (what does this mean?). If published, this will include your full peer review and any attached files.

If you choose “no”, your identity will remain anonymous but your review may still be made public.

**Do you want your identity to be public for this peer review?**  For information about this choice, including consent withdrawal, please see our Privacy Policy.

Reviewer #1: No

Reviewer #2: No

**********

# Editor report: Acceptance letter

**DOI:** 10.1371/journal.pone.0351560.r004

**Authors:** Francesco Bardozzo, Carla Pegoraro

PONE-D-25-48731R1

PLOS One

Dear Dr. Lee,

I'm pleased to inform you that your manuscript has been deemed suitable for publication in PLOS One. Congratulations! Your manuscript is now being handed over to our production team.

At this stage, our production department will prepare your paper for publication. This includes ensuring the following:

* All references, tables, and figures are properly cited

* All relevant supporting information is included in the manuscript submission,

* There are no issues that prevent the paper from being properly typeset

You will receive further instructions from the production team, including instructions on how to review your proof when it is ready. Please keep in mind that we are working through a large volume of accepted articles, so please give us a few days to review your paper and let you know the next and final steps.

Lastly, if your institution or institutions have a press office, please let them know about your upcoming paper now to help maximize its impact. If they'll be preparing press materials, please inform our press team within the next 48 hours. Your manuscript will remain under strict press embargo until 2 pm Eastern Time on the date of publication. For more information, please contact onepress@plos.org.

You will receive an invoice from PLOS for your publication fee after your manuscript has reached the completed accept phase. If you receive an email requesting payment before acceptance or for any other service, this may be a phishing scheme. Learn how to identify phishing emails and protect your accounts at https://explore.plos.org/phishing.

If we can help with anything else, please email us at customercare@plos.org.

Thank you for submitting your work to PLOS ONE and supporting open access.

Kind regards,

PLOS ONE Editorial Office Staff

on behalf of

Dr Carla Pegoraro

Staff Editor

PLOS One
